# Antioxidants-related nuclear factor erythroid 2-related factor 2 gene variants associated with HBV-related liver disease

**DOI:** 10.1186/s12935-023-02918-6

**Published:** 2023-04-16

**Authors:** Yanqiong Liu, Qiulian Wu, Fuyong Zhang, Xue Qin

**Affiliations:** 1 Key Laboratory of Clinical Laboratory Medicine of Guangxi Department of Education, Department of Clinical Laboratory, The First Affiliated Hospital of Guangxi Medical University, Nanning, Guangxi China; 2 Department of Clinical Laboratory, The People’s Hospital of Guangxi Zhuang Autonomous Region, Nanning, Guangxi China

**Keywords:** Nuclear factor erythroid 2-related factor 2, Polymorphism, Hepatocellular carcinoma

## Abstract

**Background:**

Accumulating evidence demonstrated that nuclear factor erythroid 2-related factor 2 (NRF2) expression plays a crucial role in the proliferation, invasion and metastasis of hepatocellular carcinoma (HCC). However, research on the effect of NRF2 genetic polymorphism on the development of chronic hepatitis B (CHB), HBV-related liver cirrhosis (LC) and HCC is still missing.

**Methods:**

A total of 673 individuals were included in the study and classified into four groups: 110 CHB cases, 86 LC cases, 260 HCC cases, and 217 healthy controls. ​The polymerase chain reaction-restriction fragment length polymorphism and DNA sequencing method were used to detect rs6721961 and rs6726395 polymorphisms.

**Results:**

Patients carrying the T allele in rs6721961 were at a higher risk of HCC than individuals with the G allele compared to CHB patients (OR = 1.561, 95%CI: 1.003–2.430, P = 0.048). The statistically significant differences were also found in the rs6721961 GT genotype (OR = 2.298, 95% CI: 1.282–4.119, P = 0.005) and dominant model (OR = 2.039, 95% CI: 1.184–0.510, P = 0.010). Subgroup analysis also detected a significant association between the rs6721961 T allele and the development of HCC in older subjects (≥ 50 years) (OR = 2.148, 95% CI: 1.208–3.818, P = 0.009). Statistical analysis results indicated that subjects carrying haplotype G-A had a lower risk of HCC (OR = 0.700, 95% CI: 0.508–0.965, P = 0.028).

**Conclusions:**

For the first time, our findings provide evidence that the NRF2 gene rs6721961 variation is a potential genetic marker of susceptibility to HCC.

## Introduction

Chronic liver disease and primary liver cancer, particularly hepatocellular carcinoma (HCC), are becoming more prevalent globally [1]. With approximately 906,000 new cases and 830,000 deaths, liver cancer is the sixth most frequently diagnosed cancer and the third most common cause of cancer death worldwide in 2020 [1]. China has the highest regional incidence and mortality rates, accounting for approximately 45.3% of the total caseload and 47.1% of deaths [1]. These highlight the urgent need to enhance screening methods in high-risk populations to detect susceptibility to HCC and to make an early diagnosis to prevent morbidity and mortality.

The main risk factors for HCC vary substantially by geographic region. In China, where HCC is most at risk, the main factors are chronic exposure to infected hepatitis B virus (HBV) and/or aflatoxin [2]. However, only a small percentage of people with these known risk factors end up with HCC, suggesting that genetic diversity may pave the way to HCC. Recent research has demonstrated that the pathogenesis and progression of HCC is a multi-step process involving genetic mutations and epigenetic alteration in hepatocytes [2]. The mutations in genes involved in the oxidative stress response pathway have been recognized as the major genetic aberration identified in subsets of HCC [3].

Nuclear factor erythroid 2-related factor 2 (Nrf2), encoded by the gene NRF2, is the transcription factor of the primary means of cellular defence through the mediation of antioxidant response and anti-inflammatory properties [4]. A previous study demonstrated that the induction of HBV-dependent NRF2-regulated genes might ensure the survival of the infected cell, and shape the immune response to HBV, thus promoting the establishment of infection [5]. Another study suggested that HBV up-regulated glucose-6-phosphate dehydrogenase expression by HBx-mediated activation of NRF2, thereby reprogramming the glucose metabolism in hepatocytes which may involve in the onset of HBV-related HCC [6]. Accumulating evidence has established that gene expression of NRF2 plays a key role in the pathogenesis, progression and metastasis of HCC, as well as in the regulation of metabolism in cancer cells [7–11]. ​In 2015, Zhang et al. found that the NRF2 was up-regulated in HCC and that high NRF2 expression was correlated with tumor differentiation, metastasis, and tumor size. Zhang et al. concluded that NRF2 was an independent prognostic factor in HCC patients [7]. Similar results were obtained by Shimokawa et al. in 2020 [8]. Tao et al. reported that co-expression of mutant NRF2 and mutant CTNNB1 led to clinically relevant HCC development in mice [9]. A large cohort study conducted by Iseda et al. in 2022 revealed that positive phosphorylated NRF2 expression in cancer cells was associated with poor differentiation, microscopic intrahepatic metastasis, and poor prognosis [10]. Additionally, Iseda et al. found that NRF2 located upstream of cancer metabolism and tumor immunity [10]. The expression of NRF2 is regulated by single nucleotide polymorphisms (SNPs) and other genetic elements. Recently, investigations have reported a number of SNPs (rs6721961, rs6726395, rs10506328, rs3124761, rs17458086, rs1630747, rs35652124, and so on) in NRF2 gene to the risk of various cancer including renal cell carcinoma [12, 13], pancreatic cancer [14], breast cancer [15, 16], prostate cancer [17], bladder cancer [18]. However, to the best of our knowledge, research on the role of NRF2 genetic polymorphisms in HBV-related HCC risk is still missing. Therefore, this study aims to shed the first light on the impact of NRF2 genetic polymorphisms in HBV-related HCC risk, as well as the association with chronic hepatitis B (CHB) and HBV-related liver cirrhosis (LC). This study will provide some insights into our approach to detecting susceptibility to HCC and early diagnosis of this devastating disease.

## Materials and methods

### Study design and participants

This study was a case-control association study conducted at The First Affiliated Hospital of Guangxi Medical University. The cases were admitted to the First Affiliated Hospital of Guangxi Medical University during enrollment. Included individuals were classified into four groups: CHB cases, LC cases, HCC cases, and healthy controls. All cases were tested to have a history of HBV infection and without other hepatitis viruses (hepatitis A/C/D/E virus). The diagnosis of CHB and LC was determined following the Guideline of Prevention and Treatment for Chronic Hepatitis B set by the Chinese Society of Hepatology, Chinese Medical Association and Chinese Society of Infectious Diseases, Chinese Medical Association in 2015 [19]. The diagnosis of HCC was determined following the expert consensus set by the Chinese Society of Liver Cancer (CSLC), Chinese Society of Clinical Oncology (CSCO), Liver Cancer Group, Chinese Society of Hepatology, Chinese Pathological Group of Hepatobiliary Tumors and Liver Transplantation in 2010 [20]. For all the study participants, the details of inclusion criteria and exclusion criteria have been described in detail previously [21]. The study protocol was approved by the First Affiliated Hospital of Guangxi Medical University Ethics Committee (Approval Number: 2023-E036-01).

### SNPs selection

SNPs in NRF2 genes were selected based on the data of HapMap and the following criteria: (i) minor allele frequency of ≥ 5% in the Chinese Beijing population; (ii) pairwise linkage disequilibrium (LD) with r^2^ ≥ 0.8. This SNPs selection strategy maximized the utility of the SNPs selected for our question of interest. The target SNPs were also checked in PubMed for consistency with published studies [13, 22, 23]. SNP rs6721961 is localized in the promoter region of the NRF2 gene, where can be present an adenine (A) or guanine (G). Among discovered polymorphisms, Nrf2 rs6721961 is one of the more frequently described. SNP rs6726395 is located in the first intron of the NRF2 gene, with a relatively high minor allele frequency. The association of rs6721961 and rs6726395 polymorphisms with various cancers has also been welldemonstrated. As a result, we selected two widely analyzed SNPs with satisfied criteria, rs6721961 and rs6726395, based on our questions of interest.

### Sample size estimation

The sample size in this study was estimated using the Quanto statistical program (version 1.2.4, https://bio.tools/QUANTO). An unmatched case-control design was used. A sample of diseased (cases) and non-diseased (controls) individuals were obtained from some source population; all individuals were assumed to be independent of one another [24]. The frequency of the rs6721961 T allele and rs6726395 A allele in the East Asian population are 0.216 and 0.3937, respectively (HapMap Project dbSNP database: https://www.ncbi.nlm.nih.gov/snp/rs6721961 and https://www.ncbi.nlm.nih.gov/snp/rs6726395). The inheritance model was recessive. The estimated odds ratio was set at 2.0. The desired power was 0.80 and the type I error rate was 0.05 with two sides. Based on the above parameters, at least 145 cases may have sufficient power to assess the NFR2 rs6721961 and rs6726395 polymorphisms and the risk of diseases.

### SNPs genotyping

Five mL peripheral blood samples were collected from all of the subjects in ethylenediaminetetraacetic acid (EDTA)-coated vials and stored at -20℃ until DNA extraction. Technicians performing the genotyping were blinded to the status of the cases and controls. Genomic DNA was isolated from peripheral leukocytes using the phenol-chloroform extraction method, as described previously [21]. The DNA was then quantitated by UV spectrophotometry and stored at -20°C. The polymerase chain reaction-restriction fragment length polymorphism (PCR-RFLP) method was used to amplify NRF2 (rs6721961 and rs6726395) with the following forward and reverse primers: NRF2 rs6721961 G/T, 5’- GGAGTTCGGACGCTTTGAAA − 3’ and R: 5’- GCTTCTCCGTTTGCCTTTGA − 3’; NRF2 rs6726395 A/G, 5’- CAACCAACCCTCATGAGCTG − 3’ and 5’- GCAAATGTATATGGGCGTGC − 3’. PCR products were digested by PdiI and Csp6I (Beijing Solarbio Science & Technology Co., Ltd., China) restriction enzymes, respectively. The fragments produced by digestion were 275 and 97 bp for NRF2 rs6721961 G/T, and 462 and 189 bp for NRF2 rs6726395 A/G. More than 10% of the PCR products were randomly selected for confirmation by DNA sequencing analysis by Shanghai Sangon Biological Engineering Technology & Services, yielding 100% agreement.

### Statistical analysis

Distributions of demographic and clinical characteristics between cases and controls were evaluated by the one-way ANOVA of variance analysis (for normal continuous variables) or nonparametric test (for nonnormal continuous variables), or by chi-square test (for categorical variables). Hardy-Weinberg equilibrium (HWE) in the control group was assessed by a goodness-of-fit χ2 test. Allele and genotype frequencies were compared among different groups using the χ^2^ test. The binary logistic regression model was used to obtain the estimated odds ratios (ORs) and 95% confidence intervals (CIs) after adjusting for potential confounding variables. Three different genetic models (additive model, dominant model, and recessive model) were used to comprehensively analyze the effect of tag SNPs. SHEsis software was used to estimate haplotype frequencies [25]. All statistical analyses were performed using SPSS 17.0 (IBM Inc., Chicago, IL, USA). A two-tailed p-value < 0.05 was considered statistically significant.

## Results

### Demographic and clinical characteristics of the study subjects

A total of 673 individuals were included in the study and categorized into four groups: CHB patients (CHB, n = 110), LC patients (LC, n = 86), HCC patients (HCC, n = 260) and healthy control participants (Controls, n = 217). The baseline demographic and clinical characteristics of the 673 subjects are presented in Table 1.

**Table 1:** Baseline demographics and clinical characteristics of the subjects

Parameters, n (%)	Control (n = 217)	CHB (n = 110)	LC (n = 86)	HCC (n = 260)	P value
Age (years, mean ± SD)	46.33 ± 6.855	38.21 ± 11.905	49.78 ± 11.861	49.37 ± 11.070	< 0.001
BMI (kg/m^2^, mean ± SD)	22.49 ± 3.550	22.14 ± 3.570	22.72 ± 4.348	22.32 ± 3.563	0.054
Gender					
Male	118 (54.4%)	82 (74.5%)	65 (75.6%)	231 (88.8%)	< 0.001
Female	99 (45.6%)	28 (25.5%)	21 (24.4%)	29 (11.2%)	
Ethnicity					0.064
Zhuang	110 (50.7%)	43 (39.1%)	32 (37.2%)	101 (39.3%)	
Han	96 (44.2%)	63 (57.3%)	51 (59.3)	149 (58.0%)	
Other	11 (5.1%)	4 (3.6%)	3 (3.5%)	7 (2.7%)	
smoking status					0.058
Never	145 (66.8%)	64 (58.2%)	44 (51.2%)	166 (63.8)	
Ever	72 (33.2%)	46 (41.8%)	42 (48.8)	94 (36.2%)	
Alcohol status					0.014
Never	152 (70.0%)	57 (51.8%)	56 (65.1%)	167 (64.2%)	
Ever	65 (30.0%)	53 (48.2%)	30 (34.9%)	93 (35.8%)	

*CHB* chronic hepatitis B, *LC* liver cirrhosis, *HCC* hepatocellular carcinoma, *SD* standard deviation, *BMI* body mass index

### Association between NRF2 genotypes and CHB, LC and HCC risk

The NRF2 rs6721961 and rs6726395 genotype distributions and their association with CHB, LC, and HCC risk for cases and controls are presented in Table 2. No significant deviations from the Hardy-Weinberg equilibrium (HWE) in the distributions of alleles and genotypes were observed for the tagSNPs among the control group (rs6721961, χ^2^ = 0.009, P = 0.925; rs6726395, χ^2^ = 1.475, P = 0.225), suggesting the suitability of this sample pool for genetic analysis. The minor allele frequencies (MAF) of NRF2 rs6726395 (A) and rs6721961 (T) were 19.1% and 35.5%, respectively.

**Table 2:** Genotypes distribution of NRF2 rs6721961 and rs6726395 in four groups and the risks of CHB, LC, and HCC patients

Genotypes	Controls (N = 217)	CHB (N = 110)	LC (N = 86)	HCC (N = 260)	CHB vs. controls		LC vs. controls		HCC vs. controls		HCC vs. CHB	
					OR (95% CI)^a^	*P*	OR (95% CI) ^a^	*P*	OR (95% CI)^a^	*P*	OR (95% CI)^a^	*P*
**rs6721961**												
G allele	333	178	137	387	1.00^ref^		1.00^ref^		1.00^ref^		1.00^ref^	
T allele	101	42	35	133	0.752 (0.477–1.186)	0.220	0.830 (0.525–1.314)	0.427	1.192 (0.858–1.657)	0.295	**1.561 (1.003–2.430)**	**0.048**
GG	128	75	52	143	1.00^ref^		1.00^ref^		1.00^ref^		1.00^ref^	
GT	77	28	33	101	0.701 (0.390–1.260)	0.235	1.073 (0.613–1.876)	0.806	1.402 (0.913–2.153)	0.123	**2.298 (1.282–4.119)**	**0.005**
TT	12	7	1	16	0.685 (0.222–2.114)	0.511	0.185 (0.023–1.518)	0.116	1.042 (0.447–2.428)	0.925	1.129 (0.390–3.267)	0.823
TT + GT vs.GG^b^					0.670 (0.366–1.227)	0.195	0.894 (0.484–1.648)	0.719	1.443 (0.908–2.292)	0.121	**2.039 (1.184–0.510)**	**0.010**
TT vs. GG + GT^c^					0.899 (0.286–2.824)	0.856	0.224 (0.028–1.829)	0.163	0.953 (0.408–2.224)	0.911	0.707 (0.237–2.109)	0.534
**rs6726395**												
G allele	258	139	111	324	1.00^ref^		1.00^ref^		1.00^ref^		1.00^ref^	
A allele	176	81	61	196	0.817 (0.557–1.197)	0.299	0.839 (0.570–1.234)	0.371	0.966 (0.725–1.289)	0.816	1.033(0.707–1.508)	0.868
GG	81	41	32	103	1.00^ref^		1.00^ref^		1.00^ref^		1.00^ref^	
AG	96	57	47	118	1.098 (0.615–1.959)	0.751	1.315 (0.745–2.321)	0.344	1.065 (0.690–1.646)	0.776	0.768(0.441–1.336)	0.350
AA	40	12	7	39	0.542 (0.230–1.277)	0.161	0.473 (0.185–1.211)	0.119	0.891 (0.494–1.606)	0.701	1.395(0.577–3.369)	0.460
AA + AG vs. GG^b^					1.107 (0.604–2.029)	0.743	1.128 (0.612–2.076)	0.700	0.858 (0.540–1.362)	0.515	0.859 (0.504 − 0.464)	0.577
AA vs. AG + GG^c^					0.519 (0.235–1.148)	0.105	0.448 (0.185–1.086)	0.075	0.867 (0.499–1.506)	0.611	1.726 (0.742–4.019)	0.205

CHB, chronic hepatitis B; LC, liver cirrhosis; HCC, hepatocellular carcinoma

^a^ Adjusted for gender, age, BMI, ethnicity, smoking and alcohol status

^b^Dominant model

^c^Recessive model

Boldfaced values indicate a significant difference

We investigated the association of these SNPs with CHB, LC, and HCC risk using a logistic regression model that controls for potential confounding factors including age, gender, BMI, ethnicity, smoking and alcohol status. There was no statistically significant association between NRF2 rs6721961 and rs6726395 and the risk of CHB, LC, and HCC when using health control as references in the entire population (Table 2).

We also analyzed the association between the NRF2 polymorphism and the risk of HCC and LC using CHB patients as references. Individuals carrying the T allele in rs6721961 were at a higher risk of HCC than subjects with the G allele compared to CHB patients (adjusted OR = 1.561, 95%CI: 1.003–2.430, P = 0.048) (Table 2). A similar relationship existed between rs6721961 GT genotype and risk of HCC as compared to the CHB group (adjusted OR = 2.298, 95% CI: 1.282–4.119, P = 0.005) (Table 2). The statistically significant difference was also found using a dominant model (adjusted OR = 2.039, 95% CI: 1.184–0.510, P = 0.010) (Table 2), suggesting that SNP rs6721961 might be associated with HCC progression. There was no statistically significant association between the NRF2 SNPs and LC risk using CHB patients as reference (data not shown).

### Haplotype analysis

​Haplotypes of NRF2 were constructed for CHB, LC and HCC patients and controls. Four major haplotypes were identified; the most frequent haplotype in controls and cases was haplotype G-G (Table 3). Statistical analysis results indicated that patients carrying haplotype G-A (rs6721961–rs6726395, in NRF2) had a lower risk of HCC (OR = 0.700, 95% CI: 0.508–0.965, P = 0.028) (Table 3).

**Table 3:** Haplotype frequencies analysis of *NRF2* and the risk of CHB, and LC, and HCC.

Haplotype	Controls (freq)	CHB (freq)	LC (freq)	HCC (freq)	CHB vs. controls			LC vs. controls			HCC vs. controls	
					OR (95% CI)	*P*_OR_		OR (95% CI)	*P*_OR_		OR (95% CI)	*P*_OR_
G-A	0.226	0.218	0.182	0.170	0.953 (0.645–1.410)	0.810		0.762 (0.487–1.193)	0.234		**0.700 (0.508–0.965)**	**0.028**
G-G	0.541	0.591	0.614	0.575	1.226 (0.883–1.703)	0.223		1.351 (0.942–1.937)	0.102		1.145 (0.885–1.479)	0.302
T-A	0.180	0.150	0.173	0.207	0.809 (0.519–1.261)	0.349		0.953 (0.599–1.518)	0.840		1.195 (0.864–1.652)	0.281
T-G	0.053	0.040	0.031	0.048	0.750 (0.340–1.656)	0.476		0.567 (0.217–1.483)	0.242		0.907 (0.508–1.620)	0.742

*freq* frequency, *CHB* chronic hepatitis B, *LC* liver cirrhosis, *HCC* hepatocellular carcinoma

Boldfaced values indicate a significant difference.

### Effects of selected NRF2 variables in different subgroups and their association with CHB, LC, and HCC risk

To evaluate the effects of NRF2 polymorphism on patient characteristics (gender, age, ethnicity, smoking and alcohol status), stratification analyses of Nrf2 polymorphisms and CHB, LC, and HCC risk were performed. When we stratified our population by age, we found that only older subjects (≥ 50 years) carrying the rs6721961 T allele had a significantly increased risk of HCC with adjusted OR of 2.148 (95% CI: 1.208–3.818, P = 0.009) (data not shown). There was no evidence that rs6721961 and rs6726395 were associated with CHB, LC and HCC susceptibility among patient subgroups by gender, ethnicity, smoking and alcohol status.

## Discussion

NRF2 is a transcription factor that controls cellular adaptation/protection and up-regulates antioxidant gene expression in response to oxidative stress [4, 26]. It has been suggested that NRF2 was a potential prognostic marker and promoted proliferation and invasion in human HCC and was involved in HCC metastasis and poor prognosis [7, 8, 10]. Thus, genes involved in the expression of NRF2 in the liver, which play a role in hepatic inflammation, fibrosis, and hepatocarcinogenesis, are central to a multigenic susceptibility model. NRF2 rs6721961 G > T polymorphism is located at the regulatory region controlling Nrf2 protein basal expression and self-induction [27]. NRF2 functional polymorphisms have been linked to the risk of developing several types of tumours [12–15]. The present study examined the role of rs6721961 and rs6726395 in the NRF2 gene in patients with CHB, HBV-associated LC and HCC. Two single variants, the T allele at rs6721961 and the GT genotype, were identified as associated with the development of HCC. In addition, one haplotype of G-A (rs6721961-rs6726395) was associated with decreased risk of HCC. To the best of our knowledge, our study is the first to explore the relationship between the genetic polymorphisms in the NRF2 gene, a vital antioxidant gene, and the risk of CHB, HBV-associated LC and HCC.

Oxidative stress plays a vital role in carcinogenesis and tumour progression [3]. ​ ​Several studies have demonstrated that genetic polymorphisms of enzymes involved in the production of reactive oxygen species may have a critical part in mediating susceptibility to the onset of HCC [28–30], including two of our previous studies [21, 31]. In the study conducted in the Moroccan population (96 cases and 222controls) by Ezzikouri et al. [28], GPX1 SNP alone did not influence the risk of HCC development; however, MnSOD Ala/Ala in combination with GPx1 Leu/Leu, and MnSOD Ala/Ala in combination with CAT TT had both a pronounced risk of HCC development. Another study by Su et al. [29] suggested that ECSOD polymorphism was significantly associated with HCC risk in non-HBV carriers, but no significant association was observed between MnSOD, CAT and GPx polymorphisms and HCC susceptibility in Chinese (434 cases and 480 control). These results suggest that HCC might usually be affected by multiple genes instead of a single SNP. In contrast, in our previous study (111 CHB patients, 90 LC patients, 266 HCC patients, and 248 healthy controls), Chinese subjects with one or two T alleles of CAT rs769217 had a significantly increased risk of CHB, LC, and HCC [21].

​ So far, we acknowledge that the role of the NRF2 SNP has not been investigated concerning susceptibility to the development of HCC, but only concerning several other types of tumors (Table 4). As early as 2007, Hong et al. assessed relationships between breast cancer risk and genetic polymorphisms of NRF2 (11,108 C > T) and identified a non-significant association after adjustment for potential confounders (505 cases and 502 controls) [32]. Zhang et al. reported similar null results in 2019 concerning NRF2 rs10506328 and the risk of prostate cancer (231 cases and 382 controls) [33]. However, Hartikainen et al. reported that the NRF2 rs6721961 TT genotype was associated with an increased risk of breast cancer by 4.6 times (452 cases and 370 controls) [34]. In lung cancer, Okano et al. observed that NRF2 rs6721961 SNP showed a significant association only in female non-smokers with adenocarcinoma [35]. Another recent analysis of published genome-wide association study (GWAS) datasets failed to identify any variants in the NRF2 gene to be associated with pancreatic cancer risk [14]. In the study conducted in Serbian patients (223 cases and 336 controls) in 2021, NRF2 rs6721961 alone exerted no influence on the risk of clear cell renal cell carcinoma development, but the NRF2 rs6721961 A allele plus the SOD2 rs4880 T allele showed three-times increased risk of this disease occurrence [12]. Just recently, similar results were obtained in a study conducted on Serbian patients by Djokic et al. in 2022 [32]. They reported that NRF2 rs6721961 alone did not associate with the risk for the occurrence of prostate cancer. Conversely, combining the NRF2 rs6721961 CC genotype with the SOD2 rs4880 C allele increased the risk of prostate cancer development by 4.07 times [32]. In our present study, differing from the above reports, we found that a significant association between NRF2 rs6721961 polymorphism and risk of HCC development was not observed in the entire population but only in older subjects, with respective adjusted OR of 2.148 (95% CI: 1.208–3.818, P = 0.009). In particular, carriers of one or two of the rs6721961 T allele had a nearly two-fold increased risk of developing HCC compared to carriers of the G allele when compared to CHB patients. Our results suggest that SNP rs6721961 may be associated with the development and progression of HCC. There is no evidence that NRF2 rs6726395 is associated with susceptibility to CHB, HBV-associated LC and HCC. These inconsistent findings may be due to differences in ethnic group dependence, variability across diseases and populations, small sample sizes, or interactions with environmental factors.

**Table 4:** Studies about the association of polymorphisms in NRF2 gene with the risk of various cancer

Cancer	Reference	Population (cases)	SNP	Association
Breast cancer	Hong et al.,2007 [32]	American (505)	rs1806649	CT vs. CC: OR = 1.31; 95%CI = 0.99–1.74; P = 0.20 TT vs. CC: OR = 1.08; 95%CI = 0.66–1.77; p = 0.20
Breast cancer	Hartikainen et al., 2012 [34]	Filander (452)	rs6721961	TT vs. TG/GG: OR = 4.656; 95%CI = 1.350–16.063; P = 0.008
			rs6706649	GG vs. TT/TG: OR = 0.215; 95%CI = 0.062–0.741; P = 0.008
			rs2706110	AA vs. AG/GG: OR = 0.481; 95%CI = 0.272–0.851; P = 0.011
			rs13035806	GG vs. AG/AA: OR = 0.353, 95%CI = 0.115–1.085; P = 0.058
Lung adenocarcinoma	Okano et al., 2013 [35]	Japanese (387)	rs6721961	Gender analysis in AA genotype: OR = 3.48, 95%CI = 1.05–11.51, P = 0.041
Cholangiocarcinoma	Khunluck et al., 2014 [36]	Thailand (198)	rs6726395	GG vs. AA/AG: OR = 1.05; 95%CI = 0.64–1.71; P = 0.852
			rs2886161	TT vs. CC/CT: OR = 1.07;95%CI = 0.54–2.09; P = 0.852
			rs1806649	CT/TT vs.CC: OR = 1.23;95%CI = 0.61–2.48; P = 0.561
			rs10183914	CT/TT vs.CC: OR = 1.08; 95%CI = 0.58–2.02; P = 0.811
Bladder cancer	Reszka et al., 2014 [18]	Polish (244)	rs6721961	CA vs. CC: OR = 0.97; 95%CI = 0.64–1.49; P = 0.91 AA vs. CC: OR = 0.49; 95%CI = 0.12–1.94; P = 0.31
Pancreatic cancer	Yang et al., 2019 [14]	European (8474)	164 NRF2-related genes	No variants in the NRF2 gene to be associated with pancreatic cancer risk
Prostate cancer	Zhang et al., 2019 [33]	American (231)	rs10506328	Dominant model: OR = 1.1; 95%CI = 0.58–2.19; P = 0.777 Recessive model: OR = 1.07; 95%CI = 0.72–1.59; P = 0.748 Additive model: OR = 1.06; 95%CI = 0.78–1.44; P = 0.708
Clear cell renal cell carcinoma	Mihailovic et al., 2021 [12]	Serbian (223)	rs6721961	CA + AA vs. CC: OR = 0.692; 95%CI = 0.370–1.295, P = 0.250 NRF2 (CA + AA) + GSTP1 (Ile/Val + Val/Val) vs. GSTP1 (Ile/Ile) + NRF2 (CC): OR = 2.731; 95%CI = 1.107–6.739
Testicular germ cell tumor	Bumbasirevic et al., 2022 [37]	Serbian (88)	rs6721961	CC + CA vs. AA: OR = 1.42; 95%CI = 0.20–9.72; P = 0.719
Prostate cancer	Djokic et al., 2022 [17]	Serbia (235)	rs6721961	CC vs. CA + AA: OR = 1.28; 95%CI = 0.75–2.17; P = 0.362 SOD2 (CC + CT) + NRF2 (CC) vs. SOD2 (TT) + NRF2 (CA + AA): OR = 4.07; 95%CI = 1.09–3.03; P = 0.006

This study used CHB participants as a reference and indicated a significant association between the NRF2 rs6721961 T allele and the risk of HCC. The opposing results, using healthy individuals as a reference, was the absence of a significant association in HCC risk. A possible explanation could be that the finite sample size is insufficient to compute convincing results. The control and HCC sample sizes in the present study exceed our estimated minimum sample size. However, the number of CHB and LC patients was too small. Another potential explanation may be that it is more appropriate to select participants exposed to HBV as a control group rather than healthy subjects. Because all cases were HBV-associated LC and HBV-associated HCC. Possibly, using the CHB individuals as a reference, the significant association of the rs6721961 T allele of the NRF2 gene with the development of HCC was more convincing [5]. Consequently, it remains to be clarified whether the rs6721961 polymorphism of NRF2 is indeed associated with the occurrence of HCC.

The novel aspects of our present study are as follows. First, to our knowledge, this is the first study to estimate NRF2 genotyping in patients with CHB, HBV-associated LC, and HCC. Second, our study was well designed: we comprehensively included a wild range of HBV-related patients, from CHB to LC to HCC, which is the progression of HCC. Third, our results suggest a significant association between NRF2 polymorphisms and the development of HCC. There are several subtypes of HCC, each with different tumor progressions, clinical traits, and patient outcomes. A higher quality of life and a longer lifespan for the high risk of HCC population depend on the development of biomarkers that can help in early diagnosis or the identification of those who need earlier treatment. It appears that the NRF2 (rs6721961) gene polymorphism may be a predictor of the occurrence of HCC. This observational study is expected to provide a basis towards further in-depth investigations involving a broad population, additional molecular mechanisms, and a comparatively bigger sample of participants.

The limitations are listed below. First, the control and HCC sample size in the present study were relatively large (more than our estimated minimum sample size). However, the number of patients with CHB and LC was too small. We should give a cautious overall interpretation of results. Second, some other important SNPs could not be evaluated in this study due to the fallout of our quality control and funding limitations. Our results suggest the need for a more detailed assessment of the effects of NRF2 polymorphism in HCC development, with more SNPs and a larger sample to increase efficiency.

## Conclusion

These results, are the first, to our knowledge, to estimate genetic polymorphisms of NRF2 the risk of CHB, HBV-associated LC and HCC, and suggest the potential relevance of rs6721961 polymorphism in HCC susceptibility and the progression of this disease. Further studies with a larger population should be performed to understand if NRF2 might be considered a target for HCC diagnosis and therapeutics. It is of great interest to investigate the NRF2 gene mutations and to gain insight into the underlying molecular mechanism.

## Data Availability

The datasets used and/or analyzed during the current study are available from the corresponding author on reasonable request.

## References

[1] Sung H (2021). Global Cancer Statistics 2020: GLOBOCAN estimates of incidence and Mortality Worldwide for 36 cancers in 185 countries. CA Cancer J Clin.

[2] Muller M (2020). The landscape of gene mutations in cirrhosis and hepatocellular carcinoma. J Hepatol.

[3] Ma-On C (2017). Oxidative stress indicated by elevated expression of Nrf2 and 8-OHdG promotes hepatocellular carcinoma progression. Med Oncol.

[4] DeNicola GM (2011). Oncogene-induced Nrf2 transcription promotes ROS detoxification and tumorigenesis. Nature.

[5] Schaedler S (2010). Hepatitis B virus induces expression of antioxidant response element-regulated genes by activation of Nrf2. J Biol Chem.

[6] Liu B (2015). Hepatitis B virus stimulates G6PD expression through HBx-mediated Nrf2 activation. Cell Death Dis.

[7] Zhang M (2015). Nrf2 is a potential prognostic marker and promotes proliferation and invasion in human hepatocellular carcinoma. BMC Cancer.

[8] Shimokawa M (2020). Modulation of Nqo1 activity intercepts anoikis resistance and reduces metastatic potential of hepatocellular carcinoma. Cancer Sci.

[9] Tao J (2021). Nuclear factor erythroid 2-related factor 2 and beta-catenin coactivation in Hepatocellular Cancer: Biological and therapeutic implications. Hepatology.

[10] Iseda N (2022). Impact of nuclear factor erythroid 2-Related factor 2 in Hepatocellular Carcinoma: Cancer Metabolism and Immune Status. Hepatol Commun.

[11] Zhang C, et al. Dexmedetomidine attenuates total body Radiation-Induced Acute Liver Injury in mice through the Nrf2/HO-1 pathway. Clin Lab. 2022;68(8). 10.7754/Clin.Lab.2022.220310 10.7754/Clin.Lab.2022.220310 35975484

[12] Mihailovic S (2021). The association of polymorphisms in Nrf2 and genes involved in Redox Homeostasis in the Development and Progression of Clear Cell Renal Cell Carcinoma. Oxid Med Cell Longev.

[13] Yamaguchi Y (2019). Nrf2 gene mutation and single nucleotide polymorphism rs6721961 of the Nrf2 promoter region in renal cell cancer. BMC Cancer.

[14] Yang W (2019). Three novel genetic variants in NRF2 signaling pathway genes are associated with pancreatic cancer risk. Cancer Sci.

[15] Almeida M (2019). Prognosis of hormone-dependent breast cancer seems to be influenced by KEAP1, NRF2 and GSTM1 genetic polymorphisms. Mol Biol Rep.

[16] Al Azhary NM (2016). The role of genetic polymorphisms in Nrf2 and P73 in egyptian women with breast Cancer. Mol Biol Rep.

[17] Djokic M (2022). The association of polymorphisms in genes encoding antioxidant enzymes GPX1 (rs1050450), SOD2 (rs4880) and transcriptional factor Nrf2 (rs6721961) with the risk and development of prostate Cancer. Medicina.

[18] Reszka E (2014). Polymorphisms of NRF2 and NRF2 target genes in urinary bladder cancer patients. J Cancer Res Clin Oncol.

[19] Hou J (2017). Guideline of Prevention and Treatment for Chronic Hepatitis B (2015 update). J Clin Transl Hepatol.

[20] Cong WM (2012). Expert consensus on the scheme of pathological diagnosis of primary liver cancer. Chin Clin Oncol.

[21] Liu Y (2015). Association between catalase gene polymorphisms and risk of chronic hepatitis B, hepatitis B virus-related liver cirrhosis and hepatocellular carcinoma in Guangxi population: a case-control study. Med (Baltim).

[22] Nunes Dos Santos K, et al. Polymorphism in the promoter region of NFE2L2 gene is a genetic marker of susceptibility to Cirrhosis Associated with Alcohol abuse. Int J Mol Sci. 2019;20(14). 10.3390/ijms20143589 10.3390/ijms20143589 PMC6678089 31340446

[23] Sugitani A (2019). A polymorphism rs6726395 in Nrf2 contributes to the development of Emphysema-Associated age in smokers without COPD. Lung.

[24] Gauderman WJ (2002). Sample size requirements for association studies of gene-gene interaction. Am J Epidemiol.

[25] Shi YY (2005). SHEsis, a powerful software platform for analyses of linkage disequilibrium, haplotype construction, and genetic association at polymorphism loci. Cell Res.

[26] Taguchi K (2011). Molecular mechanisms of the Keap1–Nrf2 pathway in stress response and cancer evolution. Genes Cells.

[27] Yamamoto T (2004). Identification of polymorphisms in the promoter region of the human NRF2 gene. Biochem Biophys Res Commun.

[28] Ezzikouri S (2010). Polymorphisms in antioxidant defence genes and susceptibility to hepatocellular carcinoma in a moroccan population. Free Radic Res.

[29] Su S (2015). Genetic polymorphisms in antioxidant enzyme genes and susceptibility to hepatocellular carcinoma in chinese population: a case-control study. Tumour Biol.

[30] Ma N (2019). Oxidative stress-related gene polymorphisms are Associated with Hepatitis B Virus-Induced Liver Disease in the Northern Chinese Han Population. Front Genet.

[31] Lao X (2015). Association of paraoxonase 1 gene polymorphisms with the risk of Hepatitis B Virus-related Liver Diseases in a Guangxi Population: a case-control study. Med (Baltim).

[32] Hong CC (2007). Genetic variability in iron-related oxidative stress pathways (Nrf2, NQ01, NOS3, and HO-1), iron intake, and risk of postmenopausal breast cancer. Cancer Epidemiol Biomarkers Prev.

[33] Zhang Z (2019). Polymorphisms in oxidative stress pathway genes and prostate cancer risk. Cancer Causes Control.

[34] Hartikainen JM (2012). Genetic polymorphisms and protein expression of NRF2 and sulfiredoxin predict survival outcomes in breast cancer. Cancer Res.

[35] Okano Y (2013). SNP (-617 C > A) in ARE-like loci of the NRF2 gene: a new biomarker for prognosis of lung adenocarcinoma in japanese non-smoking women. PLoS ONE.

[36] Khunluck T (2014). Association of NRF2 polymorphism with cholangiocarcinoma prognosis in Thai patients. Asian Pac J Cancer Prev.

[37] Bumbasirevic U, et al. The polymorphisms of genes encoding Catalytic antioxidant proteins modulate the susceptibility and progression of testicular germ cell tumor. Cancers (Basel). 2022;14(4). 10.3390/cancers14041068 10.3390/cancers14041068 PMC8870690 35205816

